# Analysis between Helicobacter pylori infection and hepatobiliary diseases

**DOI:** 10.3389/fcimb.2025.1477699

**Published:** 2025-03-07

**Authors:** Zhenjun Yu, Jie Chen, Mengdie Chen, Qiaoling Pan, Yaojian Shao, Xiaolong Jin, Chaohui Wang, Yuetao Zhang, Gang Lin, Ping Feng, Xiaosheng Teng

**Affiliations:** ^1^ Department of Gastroenterology, Taizhou Central Hospital (Taizhou University Hospital), Taizhou, Zhejiang, China; ^2^ Department of Endoscopy Center, Taizhou Central Hospital (Taizhou University Hospital), Taizhou, Zhejiang, China; ^3^ Department of Endocrinology, Taizhou Central Hospital (Taizhou University Hospital), Taizhou, Zhejiang, China

**Keywords:** Helicobacter pylori, fatty liver, gallstone, gallbladder polyp, cholesterol crystal

## Abstract

**Objective:**

Helicobacter pylori (*H. pylori*) represents a significant chronic health concern, affecting approximately half of the global population. While *H. pylori* infection has been closely linked to numerous extradigestive diseases, the relationship between *H. pylori* and lesions in the gallbladder and biliary tract remains under debate.

**Method:**

We retrospectively collected data from patients who underwent *H. pylori* tests at the Physical Examination Center of Taizhou Central Hospital (Taizhou University Hospital) between 2018 and 2022. Logistic regression analysis and restricted cubic spline analysis were employed to investigate the correlation between parameters and *H. pylori*. Additionally, we utilized population data from the National Health and Nutrition Examination Survey (NHANES) database as an external validation cohort.

**Results:**

A total of 30,612 patients were included in the training set, with 22,296 (72.8%) belonging to the *H. pylori* non-infection group and 8,316 (27.2%) to the *H. pylori* infection group. Compared to the non-infection group, patients in the infection group exhibited a significant decrease in albumin levels and a notable increase in total cholesterol and erythrocyte sedimentation rate levels. Furthermore, the infection group demonstrated significantly higher occurrences of gallbladder cholesterol crystals (6.0%), gallbladder polyps (20.2%), and atherosclerosis (25.6%) compared to the non-infection group, with respective rates of 5.1%, 19.1%, and 21.4% (average p < 0.05). However, no significant differences were observed between the two groups in terms of fatty liver, intrahepatic inflammation, gallstones, or cholecystitis. Additional regression analysis revealed that *H. pylori*, age, BMI, albumin, and total cholesterol were independent risk factors for the cholesterol crystals and atherosclerosis.

**Conclusion:**

*H. pylori* infection is closely associated with the gallbladder cholesterol crystals and atherosclerosis, albeit not with conditions such as fatty liver, gallbladder stones, or cholecystitis. Future research necessitates multi-center, prospective studies to corroborate these findings.

## Introduction

Helicobacter pylori (*H. pylori*), a flagellated gram-negative bacterium (Sharndama and Mba, 2022), is a highly prevalent pathogen that colonizes the human stomach. According to data collected from 73 countries between January 2000 and June 2017, the infection rate of *H. pylori* stands at 44.3%, with a higher prevalence in developing countries compared to developed ones (50.8% vs. 34.7%) (Zamani et al., 2018). It is well-established that *H. pylori* infection suppresses gastric acid secretion and triggers chronic inflammation of the gastric mucosa, subsequently altering the gastric microenvironment and leading to extensive modifications in the gastric microbiota. This infection is intimately linked to various gastrointestinal diseases, including chronic gastritis, peptic ulcers, gastric polyps or atypical hyperplasia, gastric cancer, and mucosa-associated lymphoid tissue lymphoma (Makola et al., 2007). Furthermore, recent studies have revealed a strong association between *H. pylori* and a range of disorders beyond the digestive tract (Santos et al., 2020), encompassing diabetes mellitus (DM), non-alcoholic fatty liver disease (NAFLD), osteoporosis, Alzheimer’s disease, and autoimmune thyroid disease (Upala et al., 2016).

Multiple studies have reported an association between *H. pylori* and fatty liver, yet this linkage remains controversial. Amer et al (Abo-Amer et al., 2020). conducted a study on 646 patients from four university hospitals and two research centers, utilizing *H. pylori* antigen detection in stool samples. Their analysis revealed that *H. pylori* infection serves as an independent risk factor for NAFLD and exhibiting a correlation with the increasing severity of steatosis. A study conducted in China emphasized that among diabetic individuals, *H. pylori* infection indeed elevates the risk of NAFLD. Therefore, managing glucose and eradicating *H. pylori* could potentially contribute to reducing the prevalence of NAFLD (Chen et al., 2023). However, opposing viewpoints have also emerged. For instance, Liu et al. employed a bidirectional MR approach (a natural RCT) to investigate the potential link between *H. pylori* and NAFLD, leveraging publicly accessible large-scale GWAS data. Nevertheless, their findings did not yield a significant causal relationship (Liu et al., 2022). Additionally, there have been reports linking *H. pylori* to cholelithiasis, cholecystitis, and gallbladder polyps (Cen et al., 2023; Lim et al., 2023). Due to the limited availability of literature, the exact association between *H. pylori* and gallbladder/biliary tract pathologies remains unclear.

To further assess the association between *H. pylori* and hepatobiliary diseases, this study conducted retrospective cohorts, and comprehensively analyzed various laboratory indicators and a range of conditions, including fatty liver, intrahepatic inflammation, gallstones, cholecystitis, gallbladder cholesterol crystals, and gallbladder polyps.

## Materials and methods

### Subjects and data collection

**Figure 1:**
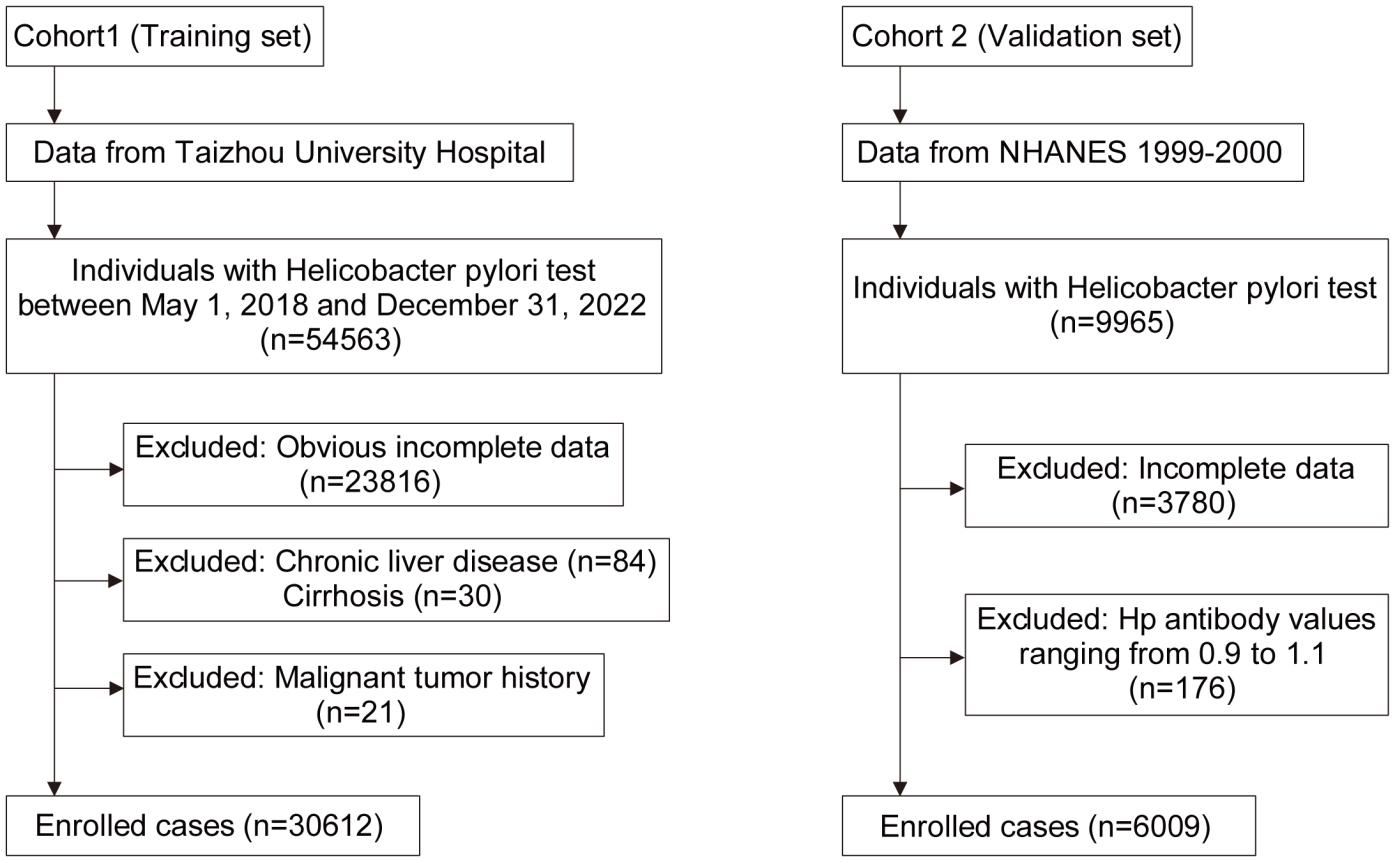
Flowchart of the physical examination population and NHANES population.

In the training set, we gathered comprehensive data on 54,563 individuals who underwent *H. pylori* testing (C13/C14) at the Physical Examination Center of Taizhou Central Hospital (Taizhou University Hospital) between May 1, 2018, and December 31, 2022. This compilation encompassed information on patients’ age, gender, height, weight, blood pressure, as well as laboratory indicators, such as triglycerides (TG), total cholesterol (TC), glucose (GLU), glycosylated hemoglobin (HbA1c), albumin (ALB), creatinine (Cr), uric acid (UA), alanine aminotransferase (ALT), aspartate aminotransferase (AST), white blood cells (WBC), mean corpuscular volume (MCV), hemoglobin (Hb), platelets (PLT), and erythrocyte sedimentation rate (ESR). During screening, 233,816 patients with extensive missing data were excluded from the analysis. Furthermore, 84 patients with chronic liver disease, 30 patients with cirrhosis, and 21 patients with a history of malignant tumors were also excluded, ultimately leading to the inclusion of 30,612 patients in our analysis (**Figure 1**). It is noteworthy that all research procedures adhered strictly to the ethical principles outlined in the Declaration of Helsinki, and the study received approval from the ethics committees of all participating institutions.

### Collection of clinical indicators

Obtained the patient’s age and gender, and measured their diastolic blood pressure (DBP), systolic blood pressure (SBP), height, and weight while they were in a calm and relaxed state. After an overnight fasting period of at least 8 hours, ultrasonography of the hepatobiliary system and arteries was performed, and venous blood samples were collected for laboratory parameter analysis. Additionally, a glycated hemoglobin analyzer was used to measure HbA1c levels.

### Detection of *H. pylori*

*H. pylori* is detected through the C13 or C14 urea breath test (Fischbach and Malfertheiner, 2018). Initially, a breath sample is collected from an individual in a fasting state. Subsequently, the individual orally administers the C13 capsule and waits for 30 minutes before collecting another breath sample to complete the C13 test. Alternatively, the individual orally ingests the C14 urea capsule along with warm water, waits for 15 minutes, and then blows air evenly through the tube for 1-3 minutes. Finally, the gas collection card is inserted into the detector to finalize the C14 test.

### National Health and Nutrition Examination Survey (NHANES) database

The NHANES database (https://wwwn.cdc.gov/nchs/nhanes/) served as the validation set. NHANES, a public database, employs a cross-sectional, stratified, multistage probability design to capture a representative sample of the civilian, non-hospitalized population in the United States. Specifically, we chose the population from the 1999-2000 cycle, as it was the only cycle that included IgG results for *H. pylori*. Out of the total 9,965 individuals, we excluded 2,472 individuals who lacked *H. pylori* data and another 1,308 individuals with incomplete data. The ELISA optical density values for all subjects ranged from 0 to 5.73. Drawing from previous research criteria (Meier et al., 2020), we classified individuals with *H. pylori* antibody values below 0.9 as negative for *H. pylori* infection and those with values exceeding 1.1 as positive. Additionally, we excluded 176 individuals with the values falling within the range of 0.9 to 1.1, ultimately including a total of 6,009 individuals in our study (**Figure 1**).

### Statistical analysis

Continuous data are reported as mean ± standard deviation, with inter-group differences evaluated using t-tests. For data that deviate from normal distribution, they are presented as median (interquartile range), and inter-group disparities are assessed via the Mann-Whitney U test. Categorical variables are reported in terms of frequency (%), and chi-square tests are employed to assess inter-group differences. Restricted cubic spline (RCS) curves are utilized to explore the nonlinear relationship. Additionally, univariate and multivariate logistic regression analyses are conducted to identify independent risk factors, and the regression equation is constructed utilizing the forward likelihood ratio. The model’s fitting effect and clinical utility are evaluated through the receiver operating characteristic curve (ROC), area under curve (AUC), calibration curve, and decision curve analysis (DCA). A two-tailed p-value <0.05 is deemed statistically significant. Statistical analysis is performed using SPSS Statistics 22.0 (IBM Corp., Armonk, New York, USA) and R (version 4.3, Statistical Computing Foundation, Vienna, Austria, https://www.R-project.org). The rms, mice, rcssci, and nhanesR packages are utilized for comprehensive statistical analysis and graphical representation.

## Results

### Clinical characteristics of patients in the training set

Based on the results of the C13 or C14 tests, patients were categorized into two groups: the *H. pylori* infection group, consisting of 22,296 individuals (72.8%), and the *H. pylori* non-infection group, encompassing 8,316 individuals (27.2%). Upon examination of the compiled data, it was observed that a minimal portion of the information was missing (**Supplementary Figure S1**). Then the mice package was utilized to perform multiple imputation for the incomplete data points. The comparative analysis revealed that the *H. pylori* infection group exhibited significantly higher levels of age, weight, and BMI compared to the non-infection group (average p < 0.001). There were no significant differences in gender and height between the two groups (**Table 1**).

**Table 1:** Comparative analysis of clinical parameters in the total population: *H. pylori* infection group versus non-infection group.

Parameters	*H. pylori* Non-Infection Group (n=22296)	*H. pylori* infection Group (n=8316)	t/χ2/Z value	*p* value
Age (years)	43.389 ± 12.603	44.943 ± 12.442	9.630	<0.001
Gender (Male)	15820 (71.0%)	5945 (71.5%)	0.841	0.359
Height (m)	1.678 ± 0.079	1.677 ± 0.079	1.433	0.152
Weight (kg)	68.139 ± 12.644	68.708 ± 12.541	3.506	<0.001
BMI (kg/m^2^)	24.076 ± 3.474	24.325 ± 3.464	5.582	<0.001
SBP (mmHg)	123.520 ± 16.574	124.536 ± 16.988	4.688	<0.001
DBP (mmHg)	75.308 ± 11.368	76.022 ± 11.565	4.866	<0.001
TG (mmol/L)	1.830 ± 1.356	1.886 ± 1.463	3.037	0.002
TC (mmol/L)	5.167 ± 0.956	5.250 ± 0.986	6.590	<0.001
ALB (g/L)	46.556 ± 2.582	46.159 ± 2.603	11.905	<0.001
Cr (mmol/L)	71.030 ± 15.392	71.712 ± 14.327	3.511	<0.001
UA (mmol/L)	365.406 ± 94.470	364.750 ± 94.263	0.540	0.589
ALT (U/L)	28.003 ± 24.480	28.118 ± 22.950	0.373	0.709
AST (U/L)	23.844 ± 12.401	24.032 ± 11.530	1.205	0.228
WBC (×10^9^/L)	6.093 ± 1.539	6.309 ± 1.530	10.961	<0.001
MCV (fL)	91.221 ± 5.009	91.269 ± 5.094	0.732	0.464
Hb (g/L)	149.301 ± 15.307	149.128 ± 15.687	0.874	0.382
PLT (×10^9^/L)	228.219 ± 50.611	229.914 ± 51.010	2.602	0.009
ESR (mm/H)	7 (2, 13)	7 (2, 14)	4.500	<0.001
GLU (mmol/L)	4.942 ± 1.090	5.040 ± 1.281	6.160	<0.001

DBP, diastolic blood pressure; SBP, systolic blood pressure; TG, triglycerides; TC, total cholesterol; ALB, albumin; Cr, creatinine; UA, uric acid; ALT, alanine aminotransferase; AST, aspartate aminotransferase; WBC, white blood cells; MCV, mean corpuscular volume; Hb, hemoglobin; PLT, platelets; ESR, erythrocyte sedimentation rate; GLU, glucose.

The blood tests revealed significantly higher levels of TG, TC, Cr, Glu, WBC, and PLT in the *H. pylori* infection group compared to the non-infection group (average p < 0.01). On the contrary, the ALB level in the infection group was significantly lower (p < 0.01). The ESR levels did not adhere to a normal distribution, with the Mann-Whitney U test revealing a significantly elevated ESR level in the infection group (p < 0.001). However, no significant differences were observed in other laboratory parameters, including UA, ALT, and AST, between the two groups (**Table 1**).

The ultrasound features of coarse or dense liver echo were utilized to assess the intrahepatic inflammation, while gallbladder wall thickening or roughness were utilized to assess the cholecystitis. Further comparison revealed significantly higher occurrences of fatty liver (42.4%), gallbladder cholesterol crystals (6.0%), gallbladder polyps (20.2%), and atherosclerosis (25.6%) in the *H. pylori* infection group compared to the non-infection group, with respective rates of 41.1%, 5.1%, 19.1%, 21.4% (average p < 0.01). However, no significant differences were observed between the two groups in terms of other features, including intrahepatic inflammation, gallstones, cholecystitis (**Table 2**).

**Table 2:** Comparative analysis of ultrasonic features in the total population: *H. pylori* infection group versus non-infection group.

Parameters	*H. pylori* Non-Infection Group (n=22296)	*H. pylori* infection Group (n=8316)	χ2 value	*p* value
Fatty liver	9155 (41.1%)	3525 (42.4%)	4.396	0.036
Dense liver echoes	2178 (9.8%)	797 (9.6%)	0.235	0.628
Cholesterol crystal	1126 (5.1%)	498 (6.0%)	10.613	0.001
Gallstone	992 (4.4%)	367 (4.4%)	0.019	0.892
Rough gallbladder wall	5130 (23.0%)	1948 (23.4%)	0.590	0.442
Gallbladder polyps	4261 (19.1%)	1677 (20.2%)	4.311	0.038
angiosclerosis	4775 (21.4%)	2126 (25.6%)	59.705	<0.001

### Correlation between *H. pylori* infection and metabolic abnormalities

**Figure 2:**
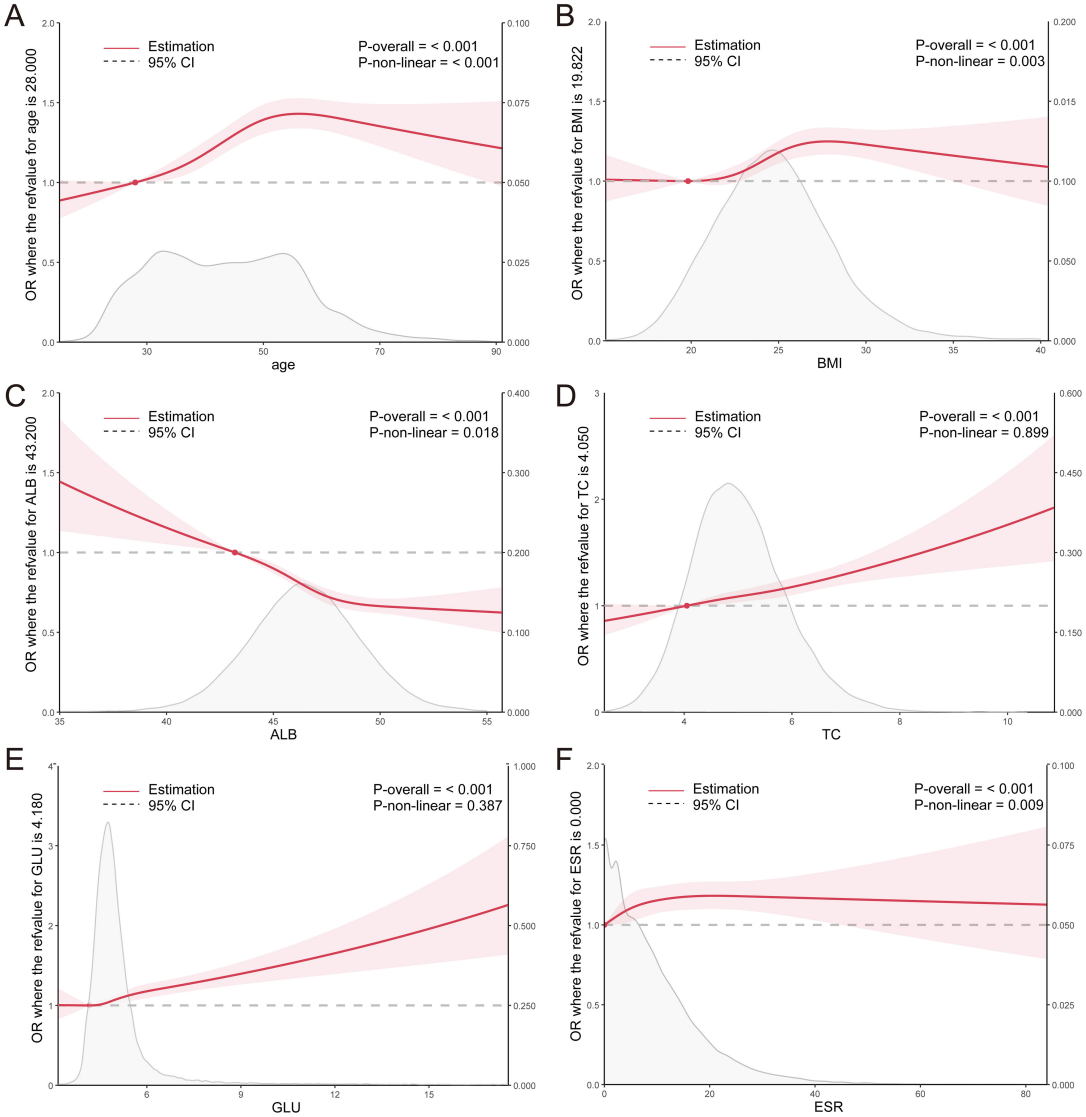
**(A–F)** RCS curves for clinical parameters and the OR value of *H. pylori* infection in the total population. ALB, albumin; TC, total cholesterol; GLU, glucose; ESR, erythrocyte sedimentation rate.

Logistic regression and RCS were employed to assess the association between the aforementioned parameters and *H. pylori*. The analysis revealed that individuals aged below 56 years exhibited an increasing risk of *H. pylori* infection with advancing age, whereas those over 56 years displayed a decreasing risk. Within the BMI range of 19.8-27.8, a significant elevation in the risk of *H. pylori* infection was observed with a rise in BMI index, whereas the risk decreased in individuals with a BMI exceeding 27.8. Notably, a significant negative correlation was observed between the ALB level and *H. pylori* infection, whereas the positive correlation was evident in TC. Furthermore, within the GLU range of >4.18mmol/L, a marked increase in the risk of *H. pylori* infection was observed with elevated glucose levels (**Figure 2**). These findings suggest a potential association between *H. pylori* infection and metabolic abnormalities such as glucose, cholesterol, and albumin. Additionally, a significant increase in the risk of *H. pylori* infection was observed with an elevation in ESR levels within the ESR range of <21.1 mm/H (**Figure 2**), indicating a potential link between *H. pylori* and the inflammatory state.

**Figure 3:**
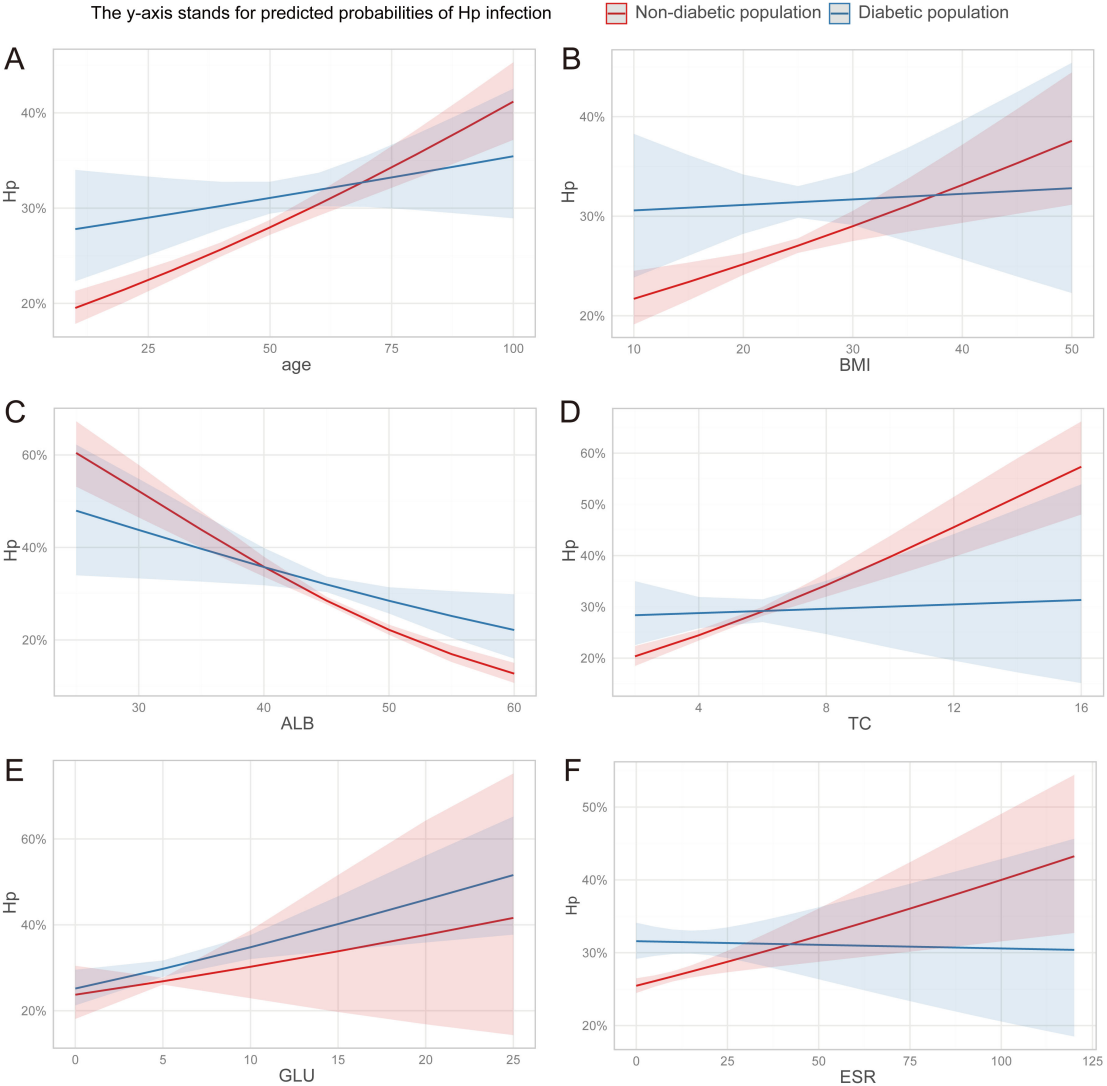
**(A–F)** The discrepancy in the association between clinical parameters and *H. pylori* infection risk among diabetic and non-diabetic individuals. ALB, albumin; TC, total cholesterol; GLU, glucose; ESR, erythrocyte sedimentation rate.

The association between *H. pylori* and metabolism may be influenced, to some extent, by abnormal glucose metabolism. Consequently, we conducted a focused analysis on the correlation between the aforementioned parameters and *H. pylori* in both diabetic and non-diabetic individuals. We further narrowed down our analysis to 18,887 individuals who had undergone HbA1c testing. Based on their medical histories and HbA1c tests, we identified 15,525 non-diabetic individuals and 3,362 diabetic individuals. Compared to the diabetic population, the trend of increasing risk of *H. pylori* infection with advancing age was more pronounced in the non-diabetic population. Similarly, the trend of elevated risk associated with decreasing ALB levels was also more evident in the non-diabetic group. More noteworthy, the significant associations between the risk of *H. pylori* infection and BMI, TC, and ESR were exclusively observed in non-diabetic individuals, excluding those with diabetes (**Figure 3**).

Consistent with the findings from the overall population, significant differences were observed in age, BMI, TC, ALB, WBC, ESR, and HbA1c levels between the *H. pylori*-infection and non-infection groups among non-diabetic individuals (**Table 3**). Similarly, the prevalence of cholesterol crystals (6.9%), gallbladder polyps (22.5%), and atherosclerosis (28.5%) was significantly higher in the infection group compared to the non-infection group, with respective rates of 5.9%, 20.5%, and 24.6% (average p<0.05) (**Table 4**). However, among diabetic individuals, due to the limited sample size, only ALB, WBC, HbA1c, and gallbladder polyps exhibited significant differences between the two groups (**Supplementary Tables S1**, **S2**).

**Table 3:** Comparative analysis of clinical parameters in the non-diabetic population: *H. pylori* infection group versus non-infection group.

Parameters	*H. pylori* Non-Infection Group (n=11381)	*H. pylori* Infection Group (n=4144)	t/χ2/Z value	*p* value
Age (years)	43.639 ± 11.965	45.313 ± 11.624	7.874	<0.001
Gender (Male)	8528 (74.9%)	3098 (74.8%)	0.048	0.826
Height (m)	1.684 ± 0.077	1.681 ± 0.077	1.735	0.083
Weight (kg)	68.234 ± 11.972	68.586 ± 11.683	1.631	0.103
BMI (kg/m^2^)	23.967 ± 3.243	24.171 ± 3.223	3.480	0.001
SBP (mmHg)	123.179 ± 16.078	123.765 ± 16.337	1.999	0.046
DBP (mmHg)	75.161 ± 11.291	75.670 ± 11.418	2.475	0.013
TG (mmol/L)	1.843 ± 1.271	1.884 ± 1.299	0.945	0.344
TC (mmol/L)	5.174 ± 0.924	5.269 ± 0.941	5.607	<0.001
ALB (g/L)	46.584 ± 2.574	46.137 ± 2.596	9.546	<0.001
Cr (mmol/L)	72.129 ± 13.775	72.514 ± 13.687	1.548	0.122
UA (mmol/L)	374.910 ± 92.910	372.140 ± 92.836	1.644	0.100
ALT (U/L)	28.139 ± 22.979	27.563 ± 21.093	1.410	0.159
AST (U/L)	23.876 ± 12.487	23.711 ± 10.718	0.754	0.451
WBC (×10^9^/L)	6.004 ± 1.516	6.246 ± 1.463	8.884	<0.001
MCV (fL)	91.604 ± 4.852	91.637 ± 5.009	0.371	0.711
Hb (g/L)	151.099 ± 14.656	150.559 ± 15.009	2.016	0.044
PLT (×10^9^/L)	225.035 ± 49.557	225.540 ± 49.176	0.562	0.574
ESR (mm/H)	7 (3, 13)	8 (3, 14)	3.540	<0.001
GLU (mmol/L)	4.748 ± 0.492	4.756 ± 0.502	0.899	0.368
HbA1c (%)	5.478 ± 0.303	5.511 ± 0.307	5.898	<0.001

DBP, diastolic blood pressure; SBP, systolic blood pressure; TG, triglycerides; TC, total cholesterol; ALB, albumin; Cr, creatinine; UA, uric acid; ALT, alanine aminotransferase; AST, aspartate aminotransferase; WBC, white blood cells; MCV, mean corpuscular volume; Hb, hemoglobin; PLT, platelets; ESR, erythrocyte sedimentation rate; GLU, glucose; HbA1c, glycosylated hemoglobin.

**Table 4:** Comparative analysis of ultrasonic features in the non-diabetic population: *H. pylori* infection group versus non-infection group.

Parameters	*H. pylori* Non-Infection Group (n=11381)	*H. pylori* Infection Group (n=4144)	χ2 value	*p* value
Fatty liver	4948 (43.5%)	1823 (44.0%)	0.328	0.567
Dense liver echoes	1214 (10.7%)	430 (10.4%)	0.271	0.603
Cholesterol crystal	669 (5.9%)	284 (6.9%)	5.013	0.025
Gallstone	525 (4.6%)	177 (4.3%)	0.822	0.365
Rough gallbladder wall	2905 (25.5%)	1072 (25.9%)	0.188	0.664
Gallbladder polyps	2333 (20.5%)	932 (22.5%)	7.253	0.007
angiosclerosis	2797 (24.6%)	1181 (28.5%)	24.532	<0.001

**Figure 4:**
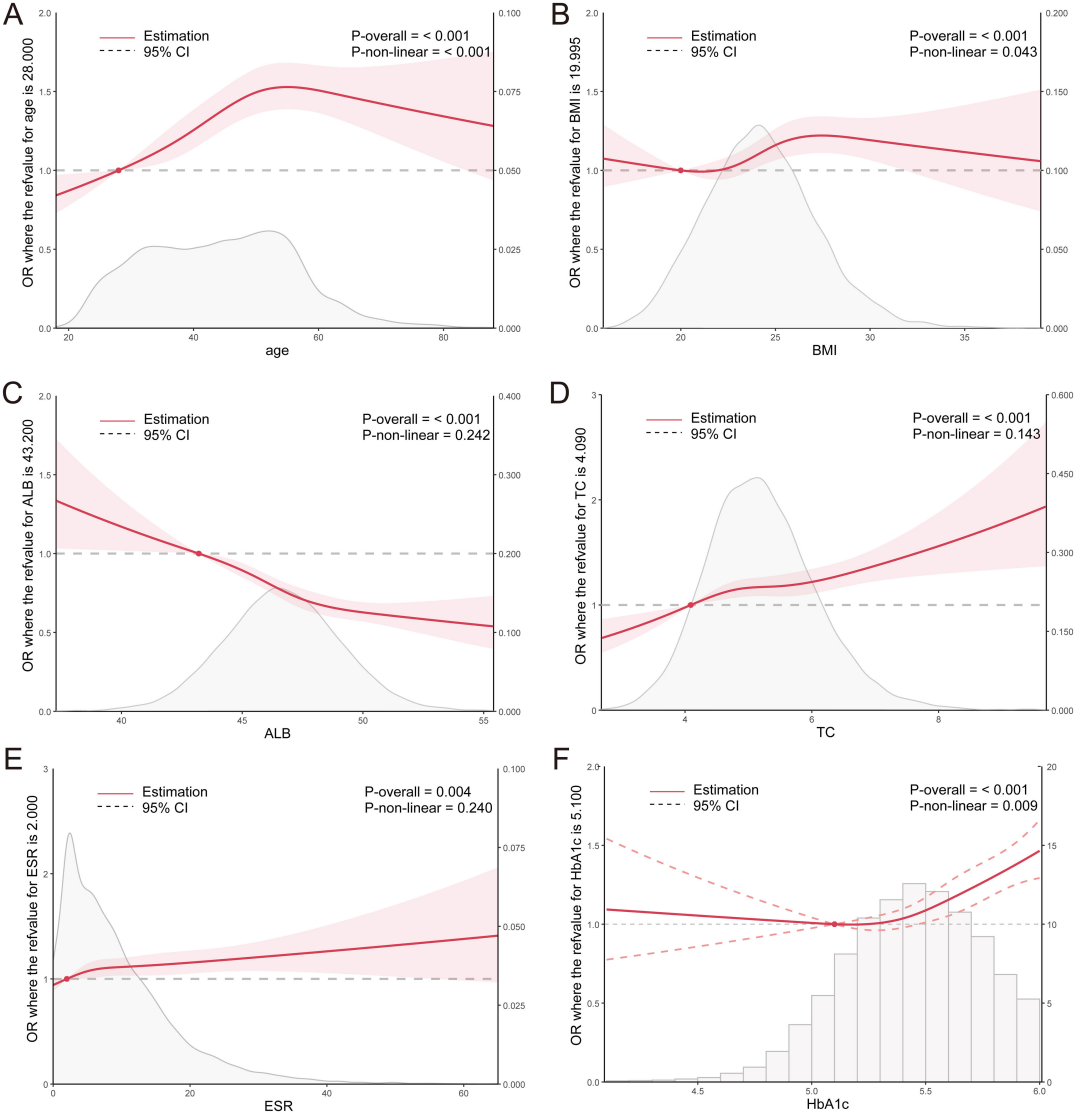
**(A–F)** RCS curves for clinical parameters and the OR value of *H. pylori* infection in the non-diabetic population. ALB, albumin; TC, total cholesterol; ESR, erythrocyte sedimentation rate; HbA1c, glycosylated hemoglobin.

Similarly, the RCS curve plotted for the non-diabetic population revealed that the risk of *H. pylori* infection increased with age among individuals younger than 55 years, whereas it decreased among those older than 55 years. Within the BMI range of 20.0-27.4, a significant elevation in the risk of *H. pylori* infection was observed with an increase in BMI. Notably, ALB levels exhibited a significant negative correlation with the risk of *H. pylori* infection, whereas TC and ESR levels demonstrated a significant positive correlation. Furthermore, within the HbA1c range of >5.1%, a marked increase in the risk of *H. pylori* infection was observed with a rise in HbA1c levels (**Figure 4**). These findings further underscore the association between *H. pylori* and abnormal metabolism of glucose, cholesterol, and albumin.

### Correlation between *H. pylori* infection and gallbladder cholesterol crystals, atherosclerosis

**Figure 5:**
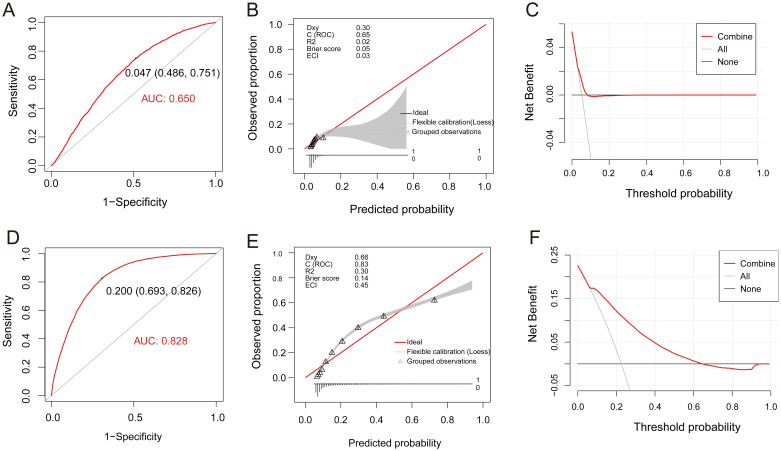
Assessment of the fit and value of the logistic regression equation model. **(A-C)**, ROC curves, calibration curve, and DCA for the model of Gallbladder Cholesterol Crystal in the total population. **(D-F)**, ROC curves, calibration curve, and DCA for the model of Atherosclerosis in the total population. ROC, receiver operating characteristic curve; DCA, decision curve analysis (DCA).

Multivariate regression analysis revealed that *H. pylori* infection, age, BMI, ALB, and TC were independent risk factors for cholesterol crystals (average p < 0.1, **Table 5**). Utilizing these parameters, a predictive model for cholesterol crystals centered on *H. pylori* was developed, formulated as follows: Y=0.112*H. pylori (yes=1, no=0) + 0.032*Age (years) + 0.049*BMI - 0.024*ALB (g/L) + 0.049*TC (mmol/L). This model exhibited an AUC value of 0.650 (95% CI: 0.644-0.655), with a statistical Z-score of 23.519 (p < 0.001). It demonstrated good sensitivity of 75.1% but relatively poor specificity of 48.6% (**Figure 5A**). Calibration curve analysis revealed a moderate alignment with the ideal state, yielding a Brier score of 0.05 (**Figure 5B**). DCA indicated moderate clinical utility for the model (**Figure 5C**). Additionally, *H. pylori* emerged as an independent risk factor for gallbladder polyps, albeit with a low predictive value (**Supplementary Table S3**, **Supplementary Figure S2**).

**Table 5:** Multivariable logistic regression for gallbladder cholesterol crystal.

Variable	B	SE	Wald χ2	p value	OR (95% CI)
*H. pylori*	0.112	0.056	4.010	0.045	1.119 (1.002-1.248)
Age	0.032	0.002	245.428	<0.001	1.033 (1.029-1.037)
BMI	0.049	0.007	43.260	<0.001	1.050 (1.035-1.066)
ALB	-0.024	0.010	5.571	0.018	0.976 (0.956-0.996)
TC	0.049	0.026	3.561	0.059	1.051 (0.998-1.106)
Constant	-4.744	0.541	76.744	<0.001	

*H. pylori*, Helicobacter pylori; TC, total cholesterol; ALB, albumin.

Subsequently, multivariate regression analysis revealed that *H. pylori* infection, age, BMI, ALB, TC, GLU, and ESR are independent risk factors for atherosclerosis (average p < 0.01, **Table 6**). Leveraging these parameters, a predictive model for atherosclerosis centered on *H. pylori* was formulated: Y=0.094* *H. pylori* (yes=1, no=0) + 0.096*Age (years) + 0.064*BMI - 0.034ALB (g/L) + 0.16*TC (mmol/L) + 0.205*GLU (mmol/L) + 0.013*ESR (mm/H). This model exhibited an AUC value of 0.828 (95% CI: 0.824-0.832), a statistical Z-score of 127.825 (p < 0.001), demonstrating high sensitivity (82.6%) and specificity (69.3%) (**Figure 5D**). Calibration curve analysis revealed a strong alignment with the ideal state, scoring a Brier of 0.14 (**Figure 5E**). Furthermore, DCA underscored the model’s significant clinical application value (**Figure 5F**).

**Table 6:** Multivariable logistic regression for atherosclerosis.

Variable	B	SE	Wald χ2	p value	OR (95% CI)
*H. pylori*	0.094	0.035	7.385	0.007	1.099 (1.027-1.176)
Age	0.096	0.002	3692.128	<0.001	1.101 (1.097-1.104)
BMI	0.064	0.005	167.942	<0.001	1.066 (1.056-1.076)
ALB	-0.034	0.007	26.640	<0.001	0.967 (0.954-0.979)
TC	0.160	0.016	94.290	<0.001	1.174 (1.136-1.212)
GLU	0.205	0.013	268.163	<0.001	1.228 (1.198-1.258)
ESR	0.013	0.002	57.027	<0.001	1.013 (1.009-1.016)
Constant	-7.845	0.345	517.456	<0.001	

*H. pylori*, Helicobacter pylori; TC, total cholesterol; ALB, albumin; GLU, glucose; ESR, erythrocyte sedimentation rate.

### Validation of the NHANES dataset

The NHANES dataset comprised 3,718 individuals in the *H. pylori* non-infection group and 2,291 in the infection group. Consistent with the findings from the training set, the *H. pylori* infection group exhibited significantly higher ages and BMI indices compared to the non-infection group (average p < 0.001). Additionally, the levels of TC, Cr, GLU, and HbA1c were notably elevated, and the levels of ALB and HDL were significantly reduced in the infection group (average p < 0.01) (**Supplementary Table S3**). Given the absence of ESR data, we resorted to the non-normally distributed C reactive protein (CRP). The Mann-Whitney U test revealed that the ESR level in the infection group was significantly elevated compared to the non-infection group (p < 0.001) (**Supplementary Table S3**).

**Figure 6:**
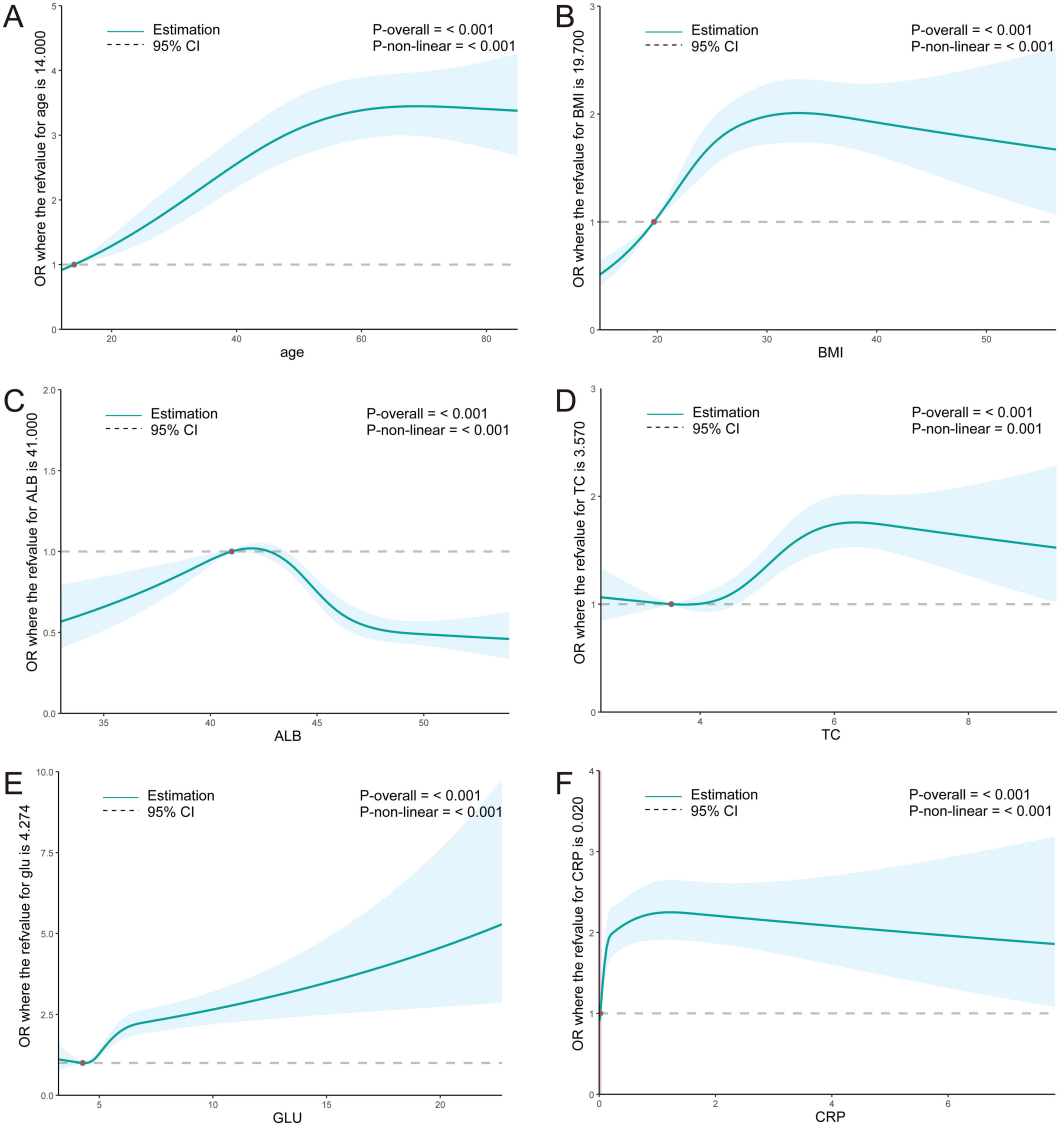
**(A–F)** RCS curves for clinical parameters and the OR value of *H. pylori* infection in the NHANES population. ALB, albumin; TC, total cholesterol; GLU, glucose; ESR, erythrocyte sedimentation rate; CRP, C reactive protein.

The RCS curve demonstrates that the risk of *H. pylori* infection rises with age among individuals younger than 55 years old. Within the BMI range of less than 28, a significant elevation in the risk of *H. pylori* infection is observed as the BMI index increases. Furthermore, in the ALB range exceeding 41g/L, there is a notable negative correlation between ALB and the risk of *H. pylori* infection. Additionally, TC and GLU levels exhibit a significant positive correlation with the risk of *H. pylori* infection. Within the CRP range below 1.2mmol/L, a marked increase in the risk of *H. pylori* infection is observed with rising CRP levels (average p<0.01) (**Figure 6**). These findings align closely with the results of the training set, further corroborating the association between *H. pylori* infection and metabolic abnormalities in glucose, cholesterol, and albumin.

## Discussion

*H. pylori* poses a significant chronic health concern, infecting approximately half of the global population (Xiong et al., 2023). Our study reveals a correlation between active *H. pylori* infection and metabolic abnormalities in glucose, cholesterol, and albumin. This association has been externally validated using the NHANES database. Previous studies have reported a higher incidence of *H. pylori* infection among patients with diabetes. For instance, the reduced secretion of hydrochloric acid in diabetic patients, often attributed to diabetes-induced dysbiosis, may create favorable conditions for the colonization of *H. pylori* (Anastasios et al., 2002). Additionally, diabetic patients with autonomic neuropathy often experience delayed gastric emptying, which not only contributes to gastrointestinal symptoms but also facilitates the occurrence of bacterial infections (Sahoo et al., 2023). A comprehensive survey of literature and meta-analysis spanning from 1996 to 2022 has revealed a trend toward more frequent *H. pylori* infection in diabetic individuals (Sahoo et al., 2023). The association between *H. pylori* and metabolism is likely influenced by abnormal glucose. To mitigate the potential confounding effect of diabetes, this study further clarified that in non-diabetic populations, ALB exhibits a significant negative correlation with *H. pylori* infection, whereas TC demonstrates a significant positive correlation. These findings align with previous research, in which Li et al. observed that for every unit increase in glucose, the prevalence of *H. pylori* increases by 6.9%, whereas for every unit increase in albumin, the prevalence decreases by 3.7% (Li et al., 2022). *H. pylori* cholesterol-α-glucosyltransferase (CGT) catalyzes the conversion of membrane cholesterol into cholesterol glucoside, which can be integrated into the bacterial membrane, thereby facilitating evasion of immune defense and colonization within the host (Hsu et al., 2021). This provides further insight into the pathophysiological mechanisms underlying the interplay between *H. pylori* and cholesterol metabolism.

In this study, both the general population and non-diabetic individuals demonstrated a close association between *H. pylori* infection and gallbladder polyps, as well as gallbladder cholesterol crystals. Notably, *H. pylori* infection emerges as an independent risk factor for the cholesterol crystals, of which the prediction model, formulated on the basis of *H. pylori* and other clinical indicators, holds significant clinical application value. Currently, there is a paucity of literature reporting on the linkage between *H. pylori* and the cholesterol crystals. We hypothesize that this association may be attributed to the impact of *H. pylori* infection on cholesterol metabolism. Furthermore, in alignment with our findings, a study encompassing 17,971 participants revealed a positive correlation between *H. pylori* and gallbladder polyps, emphasizing the likelihood of local inflammatory processes triggered by *H. pylori*, ultimately leading to an elevated incidence of gallbladder polyps (Xu et al., 2018). Nevertheless, there are also studies reporting negative outcomes, and conclusive evidence is yet to emerge to firmly establish the causal relationship between them (Lim et al., 2023).

Surprisingly, our study revealed no significant association between *H. pylori* and conditions such as fatty liver, intrahepatic inflammation, cholecystitis, and gallstones. As previously mentioned, prior research has presented contrasting views on the link between *H. pylori* and fatty liver. Similarly, conflicting findings have been reported, with one study showing that the prevalence of gallstones among *H. pylori*-positive, *H. pylori*-eradicated, and *H. pylori*-negative subjects was 9.47%, 9.02%, and 8.46% respectively. Matching analysis revealed that the incidence of gallstones was significantly lower in *H. pylori*-eradicated group, indicating a significant positive correlation between *H. pylori* infection and gallstones (Zhang et al., 2015). However, another matched case-control study focusing on Chinese patients found no significant difference in the rate of *H. pylori* infection among patients with gallstones and gallbladder polyps, suggesting that *H. pylori* infection may not be a causative factor for these conditions (Zhang et al., 2020). Our findings further underscore the uncertainty surrounding these associations and highlight the need for future multi-center, prospective studies and fundamental research to clarify these relationships.

Previous studies have extensively reported the correlation between *H. pylori* and atherosclerosis, as well as cardiovascular and cerebrovascular diseases (Saijo et al., 2005; Shi et al., 2022). Specifically, Helicobacter pylori infection has been linked to an increase in carotid intima-media thickness, particularly in CagA+ strains (Shi et al., 2022). Chen et al (Chen et al., 2024). discovered in a subgroup analysis that there exists a notable positive correlation between abdominal obesity and seropositivity for *H. pylori* antibodies in individuals less than 50 years old. These correlations are attributed to the profound influence of *H. pylori* on glucose and lipid metabolism, as well as the inflammatory status (Xie et al., 2023). *H. pylori* can trigger inflammatory reactions through diverse mechanisms. LPS, a component of the outer membrane in Gram-negative bacteria, is capable of activating the Th1 response (Candelli et al., 2023). Vacuolating cytotoxin (VacA) serves as another pivotal component in regulating the immune response to *H. pylori* infection. On the one hand, it triggers the production of cytokines, including TNF-α, MIP-1α, IL-1, IL-6, IL-10, and IL-13, by activating mast cells. On the other hand, it suppresses the proliferation and differentiation of T lymphocytes (Foegeding et al., 2016). Furthermore, a study conducted by Amedei et al. emphasized that CagA+ strains of *H. pylori*, characterized by their cytotoxin-associated gene antigen, possess a superior ability to induce the production of IL-6. Notably, IL-6 is implicated in the aging of vascular and myeloid cells, potentially leading to a mutual reinforcement that promotes atherosclerosis (Pandolfi et al., 2020). Remarkably consistent with these findings, *H. pylori* exhibited a strong positive correlation with the inflammation markers CRP and ESR in our study. Furthermore, *H. pylori* emerged as an independent risk factor for atherosclerosis, and the atherosclerotic model formulated on the basis of *H. pylori* demonstrated exceptional fitting accuracy and significant clinical application value.

Certainly, our study is not without limitations. Firstly, selection bias is inherent in our retrospective approach. Secondly, the urease breath test results solely suggest the existence of an active infection, thus rendering it impossible to ascertain the duration of the infection. It is noteworthy that cholesterol crystallization and atherosclerosis formation are protracted processes, necessitating long-term follow-up studies to yield more credible conclusions.

## Conclusion

*H. pylori* infection is associated with abnormalities in glucose, glycosylated hemoglobin, cholesterol, and albumin. Additionally, *H. pylori* poses as a risk factor for the gallbladder cholesterol crystals and atherosclerosis. However, no significant association has been observed between *H. pylori* and fatty liver, gallstones, or cholecystitis. Future multicenter, prospective, and fundamental researches are warranted to validate these findings.

## Data Availability

The raw data supporting the conclusions of this article will be made available by the authors, without undue reservation.
