# Supplementary material for: Analysis between Helicobacter pylori infection and hepatobiliary diseases
Source: Front Cell Infect Microbiol. 2025 Mar 7;15:1477699. doi: 10.3389/fcimb.2025.1477699 (PMC11926543; doi:10.3389/fcimb.2025.1477699)
Supplement: Supplementary file 1 [file DataSheet1.docx]

Supplementary Figures:


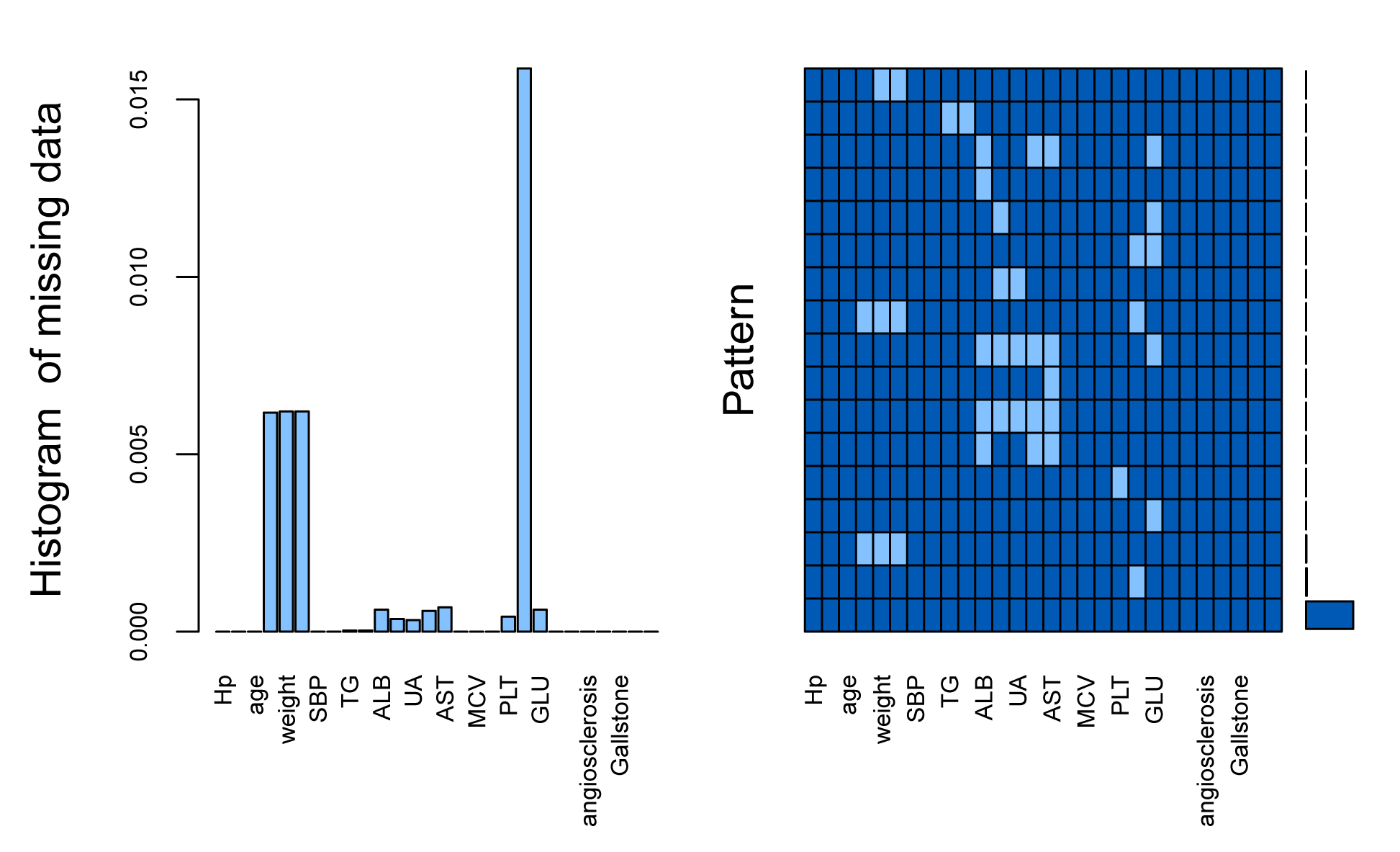


Figure S1: Display chart of missing data of total population.

Hp: Helicobacter pylori, SBP: systolic blood pressure, TG: triglycerides, ALB: albumin, UA: uric acid, AST: aspartate aminotransferase, MCV: mean corpuscular volume, PLT: platelets, GLU: glucose.


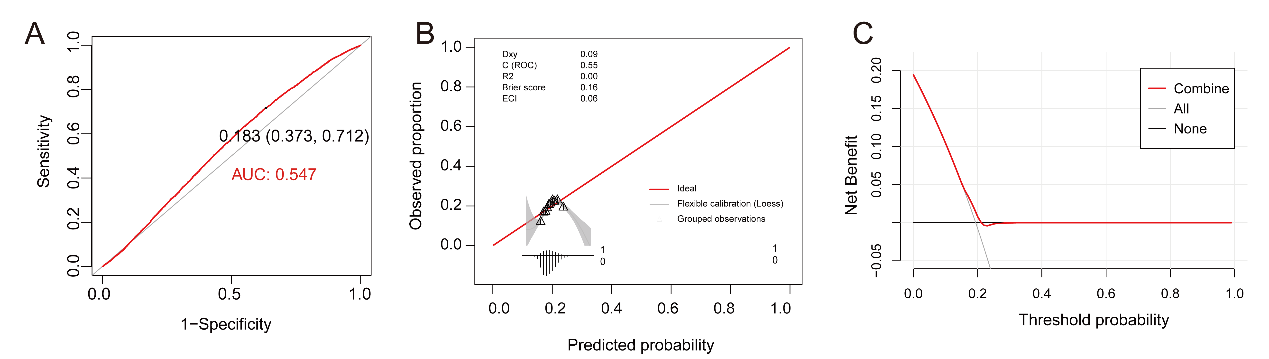


Figure S2: Assessment of the fit and value of the logistic regression equation model. A-C, ROC curves, calibration curve, and DCA for the model of gallbladder polyps in the total population.

Supplementary Tables:

Table S1: Comparative Analysis of Clinical Parameters in the Diabetic Population: H. pylori Infection Group Versus Non-infection Group

| Parameters | H. pylori Non-Infection Group (n=2305) | H. pylori Infection Group  (n=1057) | t / χ2 / Z value | *p* value |
| --- | --- | --- | --- | --- |
| Age (years) | 54.068±11.693 | 54.585±10.877 | 1.248 | 0.212 |
| Gender (Male) | 1803 (78.2%) | 817 (77.3%) | 0.362 | 0.547 |
| Height (m) | 1.672±0.080 | 1.669±0.079 | 0.951 | 0.342 |
| Weight (kg) | 71.493±12.291 | 71.314±12.158 | 0.394 | 0.694 |
| BMI (kg/m^2^) | 25.478±3.370 | 25.507±3.404 | 0.234 | 0.815 |
| SBP (mmHg) | 131.639±17.575 | 132.744±18.237 | 1.672 | 0.095 |
| DBP (mmHg) | 78.851±11.664 | 79.405±11.764 | 1.276 | 0.202 |
| TG (mmol/L) | 2.421±1.872 | 2.433±2.127 | 0.167 | 0.876 |
| TC (mmol/L) | 5.461±1.119 | 5.538±1.191 | 1.835 | 0.067 |
| ALB (g/L) | 45.899±2.584 | 45.669±2.686 | 2.367 | 0.018 |
| Cr (mmol/L) | 74.327±19.418 | 74.127±15.738 | 0.294 | 0.769 |
| UA (mmol/L) | 377.276±95.062 | 372.077±92.912 | 1.483 | 0.138 |
| ALT (U/L) | 33.468±28.635 | 31.950±23.124 | 1.636 | 0.102 |
| AST (U/L) | 26.533±14.244 | 26.203±13.011 | 0.639 | 0.532 |
| WBC (×10^9^/L) | 6.492±1.628 | 6.620±1.644 | 2.114 | 0.035 |
| MCV (fL) | 91.763±5.280 | 91.758±4.771 | 0.027 | 0.978 |
| Hb (g/L) | 150.541±13.802 | 150.817±14.640 | 0.527 | 0.598 |
| PLT (×10^9^/L) | 225.473±53.512 | 227.872±52.303 | 1.215 | 0.224 |
| ESR (mm/H) | 12 (6, 19) | 12 (6, 19) | 0.763 | 0.446 |
| GLU (mmol/L) | 6.603±2.218 | 6.856±2.461 | 2.847 | 0.004 |
| HbA1c | 6.994±1.276 | 7.105±1.410 | 2.191 | 0.029 |

DBP: diastolic blood pressure, SBP: systolic blood pressure, TG: triglycerides, TC: total cholesterol, ALB: albumin, Cr: creatinine, UA: uric acid, ALT: alanine aminotransferase, AST: aspartate aminotransferase, WBC: white blood cells, MCV: mean corpuscular volume, Hb: hemoglobin, PLT: platelets, ESR: erythrocyte sedimentation rate, GLU: glucose, HbA1c: glycosylated hemoglobin.

Table S2: Comparative Analysis of ultrasonic features in the Diabetic Population: H. pylori Infection Group Versus Non-infection Group

| Parameters | H. pylori Non-Infection Group (n=2305) | H. pylori Infection Group  (n=1057) | χ2 value | *p* value |
| --- | --- | --- | --- | --- |
| Fatty liver | 1577 (68.4%) | 703 (66.5%) | 1.208 | 0.272 |
| Dense liver echoes | 173 (7.5%) | 94 (8.9%) | 1.909 | 0.167 |
| Cholesterol crystal | 180 (7.8%) | 97 (9.2%) | 1.793 | 0.181 |
| Gallstone | 196 (8.5%) | 80 (7.6%) | 0.840 | 0.359 |
| Rough gallbladder wall | 678 (29.4%) | 323 (30.6%) | 0.453 | 0.501 |
| Gallbladder polyps | 448 (19.4%) | 246 (23.3%) | 6.514 | 0.011 |
| angiosclerosis | 1306 (56.7%) | 619 (58.6%) | 1.072 | 0.301 |

Table S3: Multivariable Logistic Regression for Gallbladder Polyps

| Variable | B | SE | Wald χ2 | p value | OR (95% CI) |
| --- | --- | --- | --- | --- | --- |
| H. pylori | 0.055 | 0.032 | 2.923 | 0.087 | 1.057 (0.992-1.126) |
| Age | 0.011 | 0.001 | 86.552 | <0.001 | 1.011 (1.009-1.013) |
| BMI | 0.011 | 0.004 | 6.240 | 0.012 | 1.011 (1.002-1.019) |
| ESR | -0.007 | 0.002 | 20.688 | <0.001 | 0.993 (0.989-0.996) |
| Constant | -2.114 | 0.111 | 363.438 | <0.001 |  |

H. pylori: Helicobacter pylori, ESR: erythrocyte sedimentation rate..

Table S4: Comparative Analysis of Clinical Parameters in the NHANES Population: H. pylori Infection Group Versus Non-infection Group

| Parameters | H. pylori Non-Infection (n=3718) | H. pylori Infection (n=2291) | t / χ2 / Z value | *p* value |
| --- | --- | --- | --- | --- |
| Age (years) | 33.965±21.356 | 44.046±22.095 | 17.397 | <0.001 |
| Gender (Male) | 1744 (46.9%) | 1148 (50.1%) | 5.822 | 0.016 |
| BMI (kg/m^2^) | 26.403±6.451 | 27.717±6.327 | 7.762 | <0.001 |
| HDL (mmol/L) | 1.330±0.369 | 1.279±0.371 | 5.181 | <0.001 |
| TC (mmol/L) | 4.848±1.113 | 5.066±1.132 | 7.334 | <0.001 |
| ALB (g/L) | 45.266±3.597 | 44.557±3.399 | 7.687 | <0.001 |
| Cr (mmol/L) | 59.958±42.114 | 63.068±45.076 | 2.706 | 0.007 |
| UA (mmol/L) | 305.541±85.602 | 315.750±89.612 | 4.410 | <0.001 |
| MCV (fL) | 89.315±5.210 | 89.429±5.473 | 0.811 | 0.417 |
| Hb (g/L) | 141.936±14.330 | 142.055±15.199 | 0.301 | 0.763 |
| PLT (×10^9^/L) | 269.388±65.712 | 267.698±65.964 | 0.967 | 0.334 |
| CRP (mmol/L) | 0.140 (0.050, 0.393) | 0.220 (0.080, 0.500) | 10.028 | <0.001 |
| GLU (mmol/L) | 5.052±1.473 | 5.464±2.164 | 8.045 | <0.001 |
| HbA1c | 5.271±0.792 | 5.603±1.190 | 11.818 | <0.001 |

HDL: high density lipoprotein, TC: total cholesterol, ALB: albumin, Cr: creatinine, UA: uric acid, MCV: mean corpuscular volume, Hb: hemoglobin, PLT: platelets, CRP: C reactive protein, GLU: glucose, HbA1c: glycosylated hemoglobin.
